# WS_2_–WC–WO_3_ nano-hollow spheres as an efficient and durable catalyst for hydrogen evolution reaction

**DOI:** 10.1186/s40580-021-00278-3

**Published:** 2021-09-20

**Authors:** Tuan Van Nguyen, Ha Huu Do, Mahider Tekalgne, Quyet Van Le, Thang Phan Nguyen, Sung Hyun Hong, Jin Hyuk Cho, Dung Van Dao, Sang Hyun Ahn, Soo Young Kim

**Affiliations:** 1 Department of Chemical and Biological Engineering, Gachon University, Seongnam-si, Gyeonggi-do 13120 Republic of Korea; 2 Department of Materials Science and Engineering, Institute of Green Manufacturing Technology, Korea University, 145 Anam-ro, Seongbuk-gu, Seoul, 02841 Republic of Korea; 3 Department of Chemical and Biological Engineering, Gachon University, Seongnam-si, Gyeonggi-do 13120 Republic of Korea

**Keywords:** TMD, TMC, TMO, WS_2_–WC–WO_3_composite, Nano hollow sphere, HER

## Abstract

Transition metal dichalcogenides (TMDs), transition metal carbides (TMCs), and transition metal oxides (TMOs) have been widely investigated for electrocatalytic applications owing to their abundant active sites, high stability, good conductivity, and various other fascinating properties. Therefore, the synthesis of composites of TMDs, TMCs, and TMOs is a new avenue for the preparation of efficient electrocatalysts. Herein, we propose a novel low-cost and facile method to prepare TMD–TMC–TMO nano-hollow spheres (WS_2_–WC–WO_3_ NH) as an efficient catalyst for the hydrogen evolution reaction (HER). The crystallinity, morphology, chemical bonding, and composition of the composite material were comprehensively investigated using X-ray diffraction, Raman spectroscopy, field emission scanning electron microscopy, and X-ray photoelectron spectroscopy. The results confirmed the successful synthesis of the WS_2_–WC–WO_3_ NH spheres. Interestingly, the presence of nitrogen significantly enhanced the electrical conductivity of the hybrid material, facilitating electron transfer during the catalytic process. As a result, the WS_2_–WC–WO_3_ NH hybrid exhibited better HER performance than the pure WS_2_ nanoflowers, which can be attributed to the synergistic effect of the W–S, W–C, and W–O bonding in the composite. Remarkably, the Tafel slope of the WS_2_–WC–WO_3_ NH spheres was 59 mV dec^−1^, which is significantly lower than that of the pure WS_2_ NFs (82 mV dec^−1^). The results also confirmed the unprecedented stability and superior electrocatalytic performance of the WS_2_–WC–WO_3_ NH spheres toward the HER, which opens new avenues for the preparation of low-cost and highly effective materials for energy conversion and storage applications.

**Supplementary Information:**

The online version contains supplementary material available at 10.1186/s40580-021-00278-3.

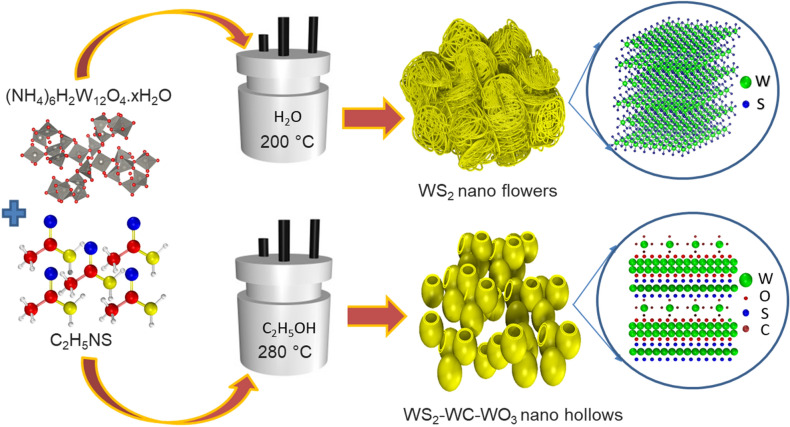

## Introduction

Over the past few decades, the over-exploitation of natural resources, such as oil, coal, and fossil gas, has been a critical challenge to humankind [1]. The consequences of the over-use of fossil fuels, including green gas emissions and global warming, have caused a severe threat to the environment and our lives [2, 3]. Therefore, scientists all over the world have endeavored to develop novel environmentally friendly and facile processes for synthesizing materials suitable for energy storage and conversion applications. Hydrogen is considered as the most efficient fuel [4–6]. The use of hydrogen gas in new technologies has increased significantly because it offers various advantages, such as poison-free gas emission and a simple and scalable production process [7, 8], which involves the application of an electric potential on electrodes in water to split it into oxygen and hydrogen. However, enhancing the efficiency of the water splitting process is a major challenge. In conventional techniques, scarce and noble materials, including platinum (Pt) and palladium (Pd), are frequently employed to produce hydrogen gas [9–11]. However, the scarcity, high cost, and poor stability of these noble materials limit their application for the production of hydrogen gas [12]. Therefore, earth-abundant metals have been extensively investigated as catalysts for the hydrogen evolution reaction (HER) [13–15].

Transition metals have partially filled d-orbitals, and hence exhibit unique mechanical and chemical properties, which have been extensively investigated [16, 17]. Interestingly, transition metal dichalcogenide (TMD) materials exhibit impressive electrocatalytic efficiency [18–20] owing to their abundant active sites, earth-abundance, and excellent stability in acidic or basic media [21]. An electrocatalyst should exhibit good conductivity improve the performance of the process [22]. However, the intrinsic conductivity of TMD materials is highly dependent on their morphology, structure, and chemical bonding. TMD materials exhibit poor intrinsic conductivity, which restricts their use in many electrochemical applications [23]. Recently, MoS_2_ and WS_2_ prepared using various techniques and procedures have been widely investigated for HER applications [24–27]. The different morphologies of WS_2_, such as nanosheets, nanoflowers (NFs), nanoparticles, nanotubes, and nano-hollow (NH), obtained using different synthesis methods showed different catalytic activities toward hydrogen generation. Nevertheless, the efficiency of these materials still needs to be improved to realize their industrial applications. Transition metal oxides (TMOs) also show various advantages, such as high intrinsic catalytic activity and a variety of desirable physical and chemical properties. The synergistic effect of WS_2_ and WO_3_ on the HER performance of their composite material has been investigated in detail [28, 29]. At the edge of the art, a new approach to improve the electrocatalytic performance of WS_2_ is to combine it with a transition metal carbide (TMC) material, such as WC or MoC [30, 31]. Transition metal carbides (TMCs) show high metallic conductivity and ceramic properties, including chemical durability, high hardness, and high melting point. Recently, TMC materials have been widely used in electrochemical applications owing to their high surface area, excellent electronic conductivity, hydrophilicity, and high chemical and mechanical durability [32, 33].

Herein, we propose a novel strategy to prepare WS_2_–WC–WO_3_ NH composite materials (WS_2_–WC–WO_3_ NH). For comparison, WS_2_ NFs were also synthesized by slightly modifying the procedure used for synthesizing the WS_2_–WC–WO_3_ NH spheres. The morphologies, structures, and chemical bonding of both the WS_2_–WC–WO_3_ NH spheres and WS_2_ NFs were investigated various techniques such as X-ray diffraction (XRD), Raman spectroscopy, field emission scanning electron microscopy (FE-SEM), and X-ray photoelectron spectroscopy (XPS), respectively. The results confirmed the successful preparation of the hybrid NH spheres of WS_2_, WC, and WO_3_. Interestingly, nitrogen was also detected in the hybrid NH spheres because it was doped in-situ into the hollow composite during the synthesis process. The high HER activity of the hybrid composite can be attributed the presence of WC, WO_3_, and nitrogen in it. Nitrogen doping significantly increased the electrical conductivity of the composite. Moreover, the synergistic effect of W–S, W–C, and W–O bonding improved the electrocatalytic performance of the WS_2_–WC–WO_3_ NH hybrid. Furthermore, the HER electrocatalytic performances of both the WS_2_ NFs and WS_2_–WC–WO_3_ NH spheres were carefully evaluated using a normal three-electrode system. The results showed that the WS_2_–WC–WO_3_ NH composite is a promising material for next-generation catalysts, which can be employed in energy conversion and storage applications.

## Experimental details

### Chemical materials

Ammonium meta-tungstate (AMT, (NH_4_)_6_H_2_W_12_O_40_) and thioacetamide (TAT, C_2_H_5_CS, 99%) were purchased from Sigma-Aldrich. Deionized (DI) water was purchased from Millipore Milli-Q system. Absolute ethanol (EtOH, C_2_H_5_OH) was obtained from Alfa Aesar. All the materials were used as received without further purification.

### Synthesis of the WS_2_–WC–WO_3_ NH sphere composite

The WS_2_–WC–WO_3_ NH sphere composite was synthesized using a conventional solvothermal technique. First, 4 g of TAT was dissolved in 20 mL EtOH by stirring at room temperature for 30 min. Then, 4 g of AMT was added to the solution and stirred continuously for another 30 min. The prepared solution was then transferred to a Teflon autoclave for 24 h at 280 °C. The autoclave was then cooled to room temperature. The resulting product was collected and washed with DI water and EtOH three times through centrifugation. Finally, the product was placed in a furnace and dried at 100 °C for 12 h to obtain the WS_2_–WC–WO_3_ NH composite. Finally, the product was dispersed in DI water by sonication and used for further measurements.

### Synthesis of the WS_2_ NFs

The WS_2_ NFs were synthesized by slightly modifying the procedure used for the synthesis of the WS_2_–WC–WO_3_ NH sphere composite. First, 4 g of TAT was dissolved in 20 mL DI water under stirring at room temperature for 30 min. To the resulting mixture, 4 g of AMT was added and the mixture was stirred continuously for another 30 min. The reaction mixture was then transferred to a Teflon autoclave for 24 h at 200 °C. The autoclave was then cooled to room temperature. The product was collected and washed with DI water and EtOH three times through centrifugation. Then, the product was placed in a furnace and dried at 100 °C for 12 h to obtain the WS_2_ NFs. Finally, the product was dispersed in DI water by sonication for further measurements.

### Materials characterization

The crystalline structures of the as-synthesized WS_2_ NFs and WS_2_–WC–WO_3_ NH spheres were analyzed using XRD (D8-Advance/Bruker-AXS). The morphology, size, and shape of the synthesized WS_2_ NFs and WS_2_–WC–WO_3_ NH spheres were analyzed using FE-SEM (SIGMA/Carl Zeiss). Raman spectroscopy (Lab RAM HR, Horiba Jobin Yvon) was performed to investigate the chemical bonding and structure of the materials. The chemical compositions of the WS_2_ NFs and WS_2_–WC–WO_3_ NH spheres were examined using XPS (Thermo Fisher Scientific, K-Alpha, USA) at a base pressure of 1 × 10^–5^ mbar and 300 K with monochromatic Mg Kα radiation (1250 eV) and a constant pass energy of 50 eV.

### Electrochemical measurements

The HER performances of the as-prepared WS_2_ NFs and WS_2_–WC–WO_3_ NH spheres were carefully evaluated using a three-electrode system in a standard 0.5 M H_2_SO_4_ solution. A carbon rod electrode and a saturated calomel electrode were used as the auxiliary and reference electrodes, respectively. A glassy carbon electrode (GCE) with a diameter of 3 mm was coated with the active material to form the working electrode. The active material was prepared by mixing 2 mg of each sample into 1 mL of DI water and 80 µL of Nafion (5%) as the stabilizer, followed by sonication for 30 min to form a homogeneous ink. Then, the homogeneous ink was deposited on the surface of the GCE and dried at 80 °C for 30 min. Linear sweep voltammetry (LSV) was performed at a scan rate of 5 mV s^−1^. To determine the double layer capacitances (C_dl_) of the samples, the measurements were performed from 0 to 0.2 V at various scan rates of 5, 10, 20, 30, 40, and 50 mV s^−1^. Electrochemical impedance spectroscopy (EIS) measurements were performed at a potential of -0.33 V vs. RHE over a frequency range of 100 kHz–0.1 Hz. All the potentials were referenced to the reversible hydrogen electrode (RHE) using the Nernst equation: E_RHE_ = E_SCE_ + E^o^_SCE_ + 0.059 pH.

## Results and discussion

**Fig. 1:**
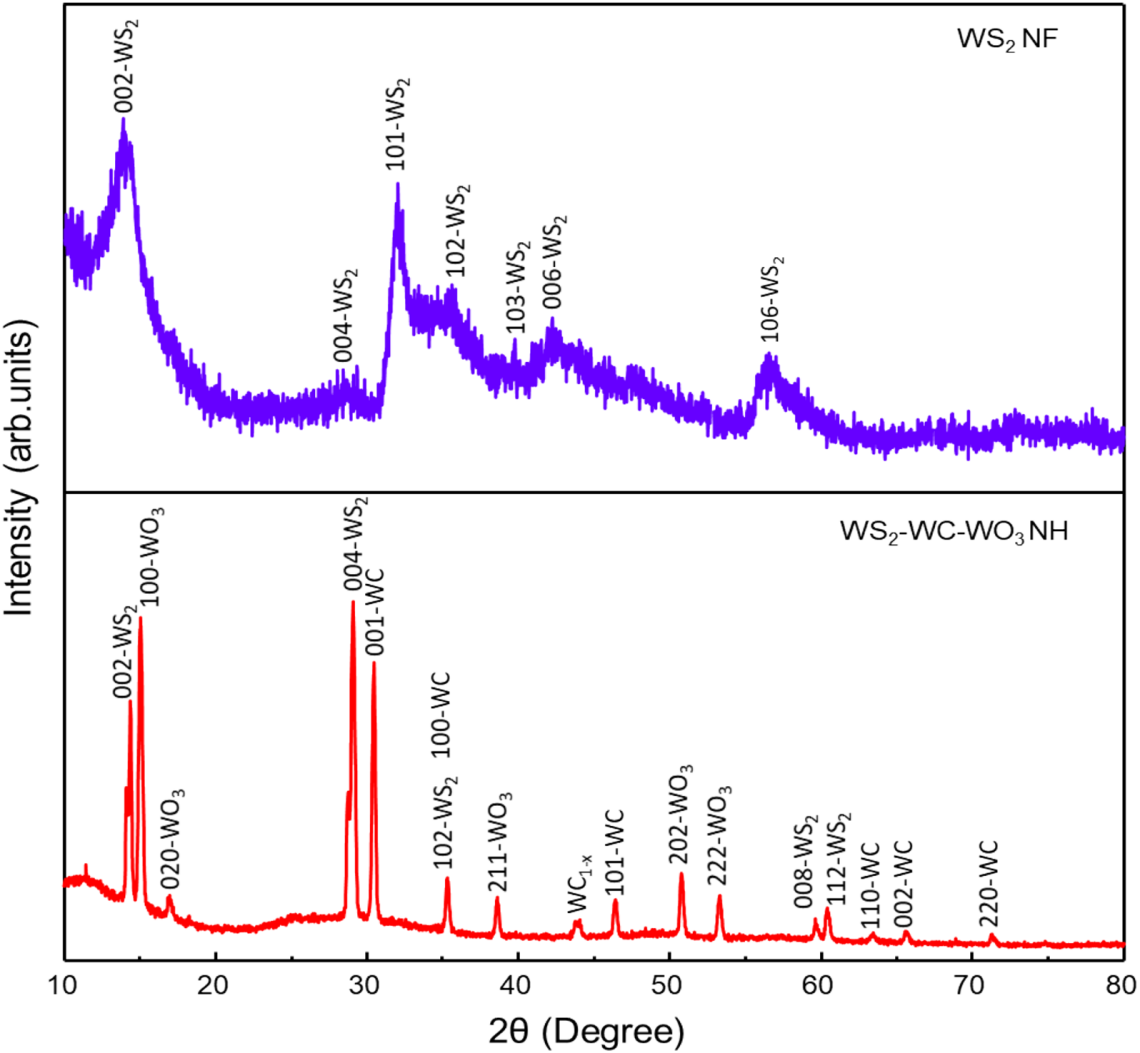
XRD patterns of the WS_2_ NFs and WS_2_/WC/WO_3_ NH spheres

The XRD patterns of the WS_2_–WC–WO_3_ NH spheres and WS_2_ NFs were obtained to analyze their structures and crystallinity. Figure 1 shows the XRD patterns of the as-prepared WS_2_ NFs and WS_2_–WC–WO_3_ NH spheres. The XRD pattern of the WS_2_ NFs showed peaks corresponding to the hexagonal phase of WS_2_ (JCPDS Card No. No. 08-0237) [34–36]. The XRD pattern of the WS_2_–WC–WO_3_ NH spheres showed numerous sharp peaks, indicating the complex growth of different elements. The peaks corresponding to WS_2_, WC, and WO_3_ could be clearly observed, indicating the coexistence of WS_2_, WC, and WO_3_ in the composite. This confirms the successful synthesis of the composites of WS_2_, WC, and WO_3_. The sharp peaks of WS_2_, which also appeared in the XRD pattern of the WS_2_–WC–WO_3_ NH spheres, were located at approximately 2θ = 14.5°, 29°, 35.5°, and 60°. The peaks located at approximately 2θ = 30.5°, 35.5°, 47°, 63°, 66°, and 71.5° indicate the presence of WC. This is consistent with the results reported previously [37]. The peak observed at 2θ = 43.5° can be attributed to the WC_1-x_ structure [38]. The peaks corresponding to WO_3_ were observed at approximately 2θ = 15°, 17°, 38°, 51°, and 53°. The results confirmed the existence of the orthorhombic structure of WO_3_ in the composite [39, 40]. The XRD pattern of the WS_2_ NFs did not show any peak corresponding to WC or WO_3_, confirming the successful preparation of pure WS_2_. The coexistence of WS_2_, WC, and WO_3_ in the WS_2_–WC–WO_3_ NH sphere composite can be attributed to the higher reaction temperature used in the synthesis process than that used in the case of WS_2_ NFs. The higher reaction temperature caused the replacement of S elements by C or O elements in the material.

**Fig. 2:**
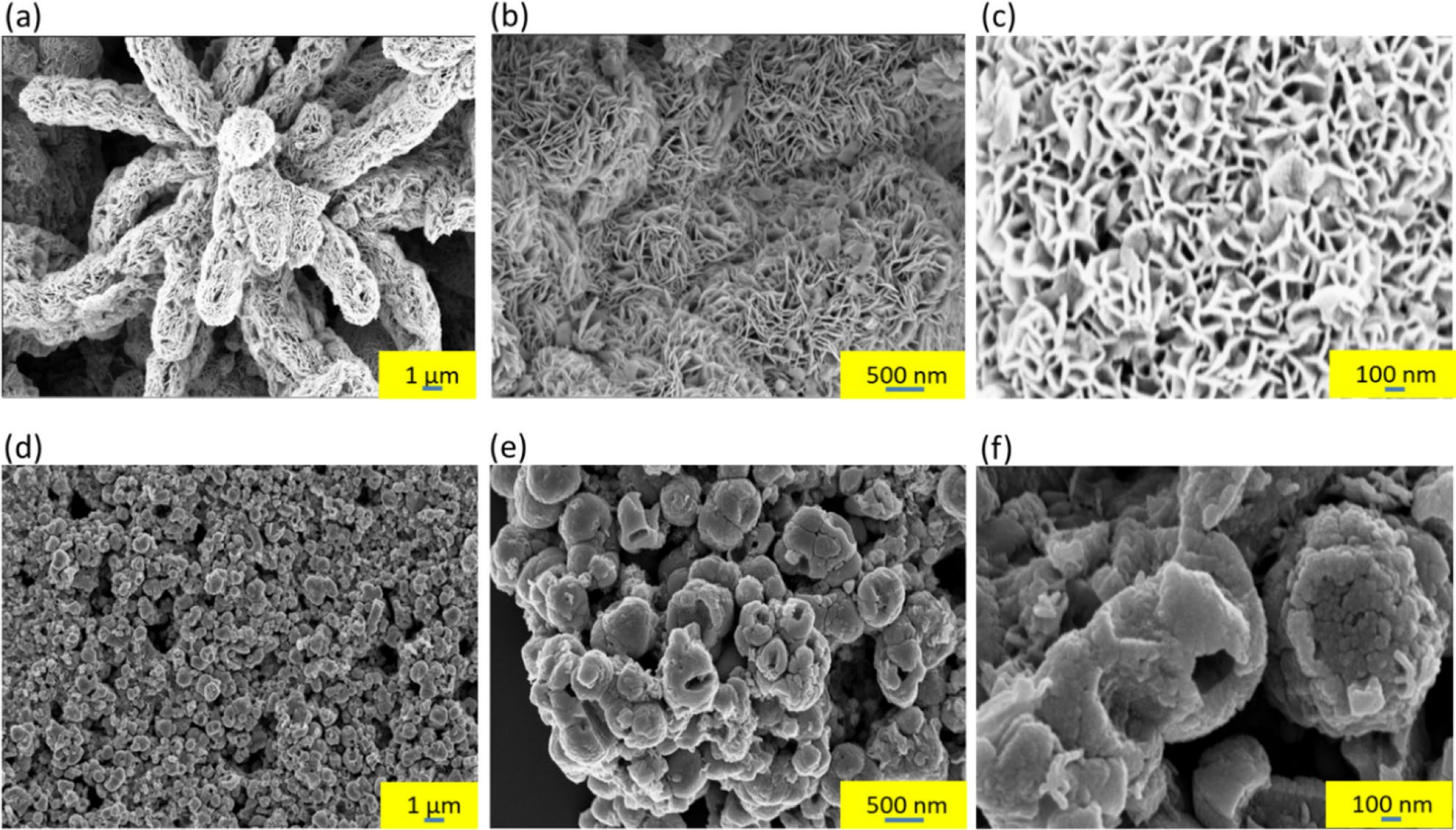
FE-SEM images of **a**–**c** the WS_2_ NFs and **d**–**f** WS_2_–WC–WO_3_ NH spheres at different scales

The morphologies of the WS_2_–WC–WO_3_ NH spheres and WS_2_ NFs were carefully observed using FE-SEM, as shown in Fig. 2. The FE-SEM images of the WS_2_–WC–WO_3_ NH spheres and WS_2_ NFs were obtained at various magnifications to clearly observe their sizes, shapes, and morphologies. Figure 2a and c show the SEM images of the highly uniform WS_2_ NFs at different scales. The thickness of the NFs ranged from 10 to 20 nm, and the distance between them was approximately 50–150 nm. In contrast, the FE-SEM images of the WS_2_–WC–WO_3_ composite showed hollow nanospheres. As can be observed from Fig. 2d–f, the diameter of these hollow nanospheres ranged from 300 to 700 nm, and the wall thickness was approximately 30–50 nm. The size of the WS_2_–WC–WO_3_ NH spheres was much smaller than that of the WS_2_ NH spheres reported in a previous study (300 nm–2 μm) [41]. This indicates that the active sites of the WS_2_–WC–WO_3_ NH spheres were significantly larger than those of the WS_2_ NFs. Interestingly, as can be observed from Fig. 2f, the surface of the WS_2_–WC–WO_3_ NH spheres was intensively and uniformly cracked. The hollow shape of the material might have been caused by the high synthesis temperature, which created high pressure inside the autoclave. The numerous cracks on the surface of the composite material can be attributed to the decomposition of TAT, in which NH_3_ and H_2_S gases were released during the sulfurization and carbonization of tungsten in AMT. This resulted in the formation of a hollow structure with numerous cracks on the surface. In addition, the nitrogen released from AMT was trapped in a hollow structure, which caused the in-situ nitrogen doping of the WS_2_–WC–WO_3_ NH composite. The EDS mapping of WS_2_–WC–WO_3_ NH composite were conducted. The EDS data of WS_2_–WC–WO_3_ NH composite was showed in the Additional file 1: Figure S1. The data confirms the well-define spatial elemental distribution for W, S, O, C, N atoms which were 35.24%, 25.32%, 19.53%, 19.41% and 0.45%, respectively. The presence of nitrogen was also detected using XPS. The empty space inside the spheres and the vast cracks on their surface contributed to the excellent electrocatalytic performance of the WS_2_–WC–WO_3_ NH composite by creating a large number of active sites in it. The hollow structure of the material also improved its stability because it prevented the depletion of materials in the electrochemical process.

**Fig. 3:**
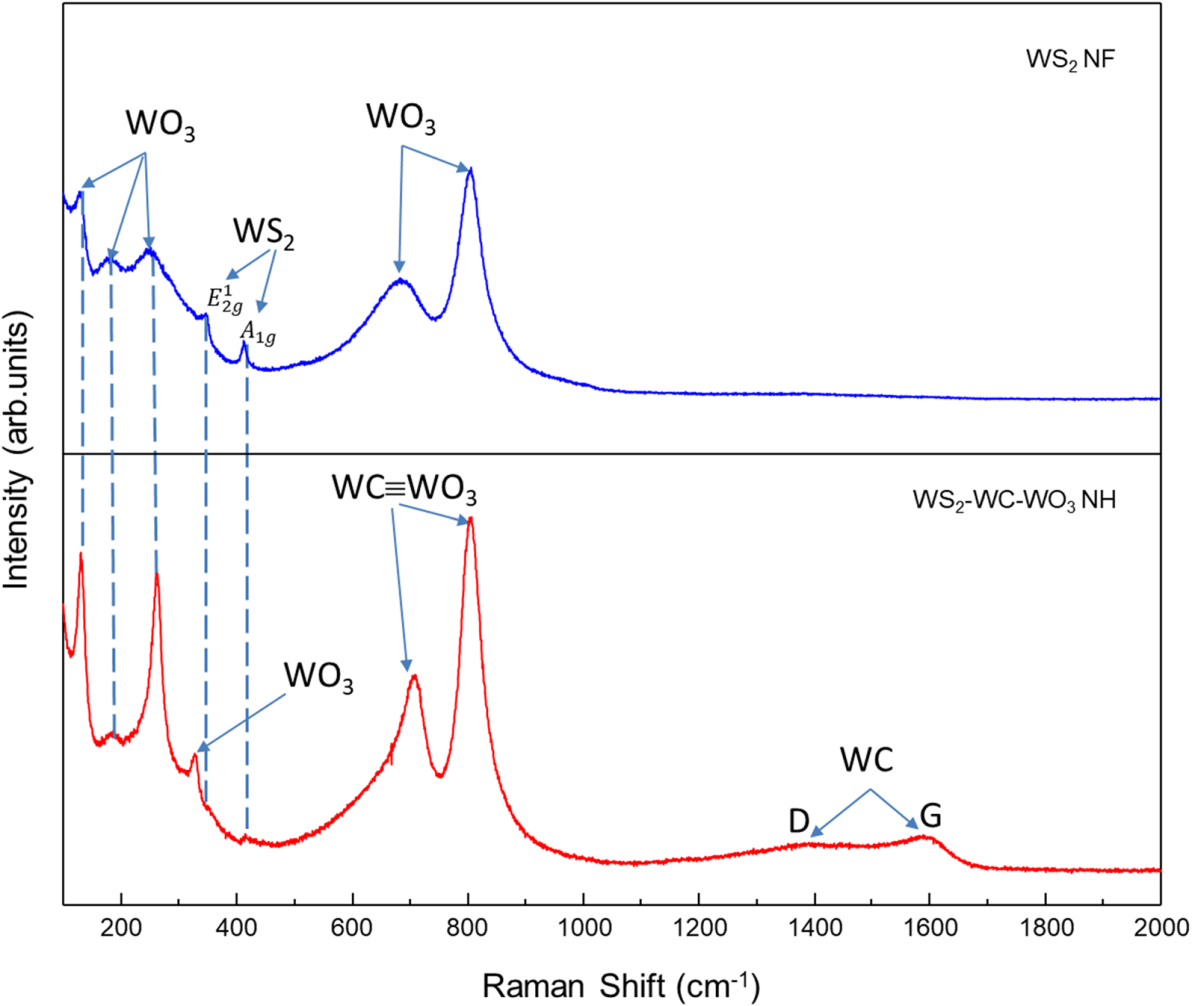
Raman spectra of the WS_2_ NFs and WS_2_–WC-–WO_3_ NH spheres

The chemical structures and bonding of the WS_2_–WC–WO_3_ NH spheres and WS_2_ NFs were further investigated by obtaining their Raman spectra in Fig. 3. The Raman spectrum of the WS_2_ NFs showed bands corresponding to the W–S bonds at 352 and 417 cm^−1^. These bonds were related to the weak van der Waals interlayer interactions affecting the intralayer bonding and lattice vibration of stacked layer crystallites [42, 43]. These bands belong to the E_2g_ mode of the W + S movement in the x–y plane and the A_1g_ mode of the S movement along the z-axis [44, 45]. The Raman spectrum of the WS_2_ NFs showed peaks corresponding to WO_3_ at 131.3, 180, 261.9, 695, and 805 cm^−1^, which resulted in the surface oxidation of WS_2_. The Raman spectrum of the WS_2_–WC–WO_3_ NH spheres showed two peaks corresponding to WS_2_ at approximately 352 and 417 cm^−1^. The Raman peaks corresponding to WO_3_ could be clearly observed at 131.3, 185, 261.9, 327, 695, and 805 cm^−1^. The sharp WO_3_ peak centered at 327 cm^−1^ marked the most significant difference between the Raman spectra of the two materials. This peak indicates the co-existence of WO_3_, WS_2_, and WC in the composite materials. The band at 695 cm^−1^ corresponds to the O–W–O stretching mode of WO_3,_ while that at 805 cm^−1^ could be ascribed to the asymmetric stretching mode of oxygen bridge (O–W–O). These O–W–O bands existed because the composite exhibited basal plane orientations that were perpendicular to the substrate surface. The Raman spectrum of the WS_2_–WC–WO_3_ NH spheres showed broad bands at approximately 1393 and 1594 cm^−1^ corresponding to WC [38]. Interestingly, the Raman peaks corresponding to WC were observed at approximately 262, 329, 710.8, and 807.2 cm^−1^. These peak positions were quite overlapping as compared to those of WO_3_ [46].

**Fig. 4:**
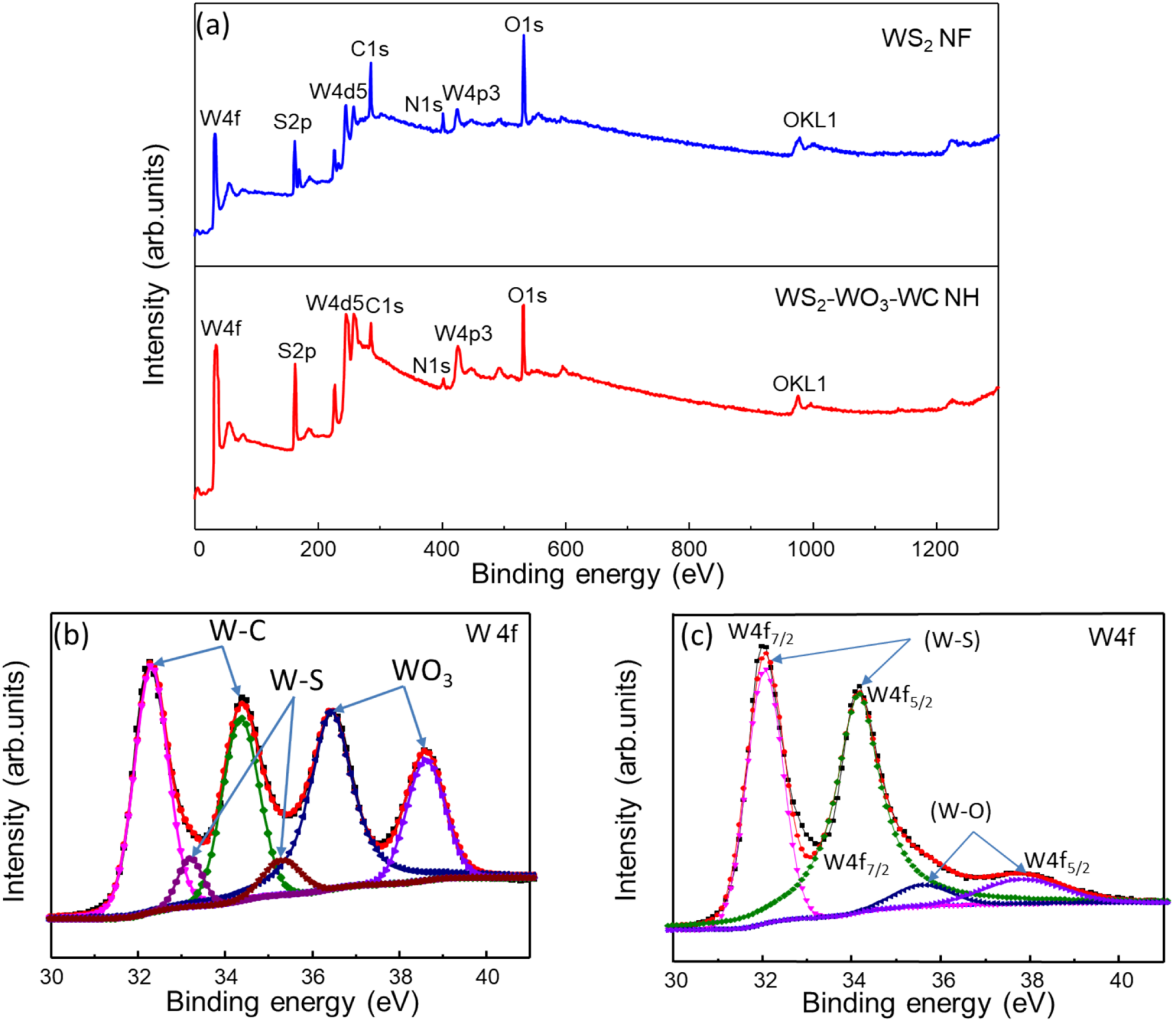
**a** XPS survey profiles of the WS_2_–WC–WO_3_ NH spheres and WS_2_ NFs, high-resolution W4f XPS profiles of the **b** WS_2_–WC–WO_3_ NH spheres and **c** WS_2_ NFs

The elemental compositions of the WS_2_ NFs and WS_2_–WC–WO_3_ NH spheres were further investigated by carrying out XPS measurements, as shown in Fig. 4. The wide XPS survey profiles of the WS_2_ NFs and WS_2_–WC–WO_3_ NH spheres are shown in Fig. 4a. As can be observed from the figure, the XPS profiles of both the WS_2_ NFs and WS_2_–WC–WO_3_ NH spheres showed nitrogen peaks, indicating that nitrogen was doped in-situ into the WS_2_ NFs and WS_2_–WC–WO_3_ NH spheres during the synthesis process. This is contrary to the results of a previous study, in which the nitrogen doping of the catalytic material was not observed [24]. The appearance of nitrogen was caused by the use of AMT as the precursor during the synthesis. The presence of nitrogen in the WS_2_–WC–WO_3_ NH composite significantly improved its conductivity and electrocatalytic performance [47]. The significant differences in the chemical composition and the W 4f binding energy levels of the WS_2_ NFs and WS_2_–WC–WO_3_ NH spheres can be clearly observed from Fig. 4b and c. As can be observed from Fig. 4b, the W 4f profile of the WS_2_–WC–WO_3_ NH spheres showed peaks corresponding to W–C, W–S, and W–O [48] because of the presence of WS_2_, WC, and WO_3_. The synergistic effect of these components improved the material characteristics, leading to a significant improvement in its HER performance as compared to that of the bare WS_2_ [49]. In Fig. 4c, The W 4f XPS profile of the WS_2_ NFs showed only the peaks corresponding to W–S and W–O, which originated from surface oxidation of WS_2_ (Fig. 4c). The peaks located at approximately 32 and 34.2 eV could be assigned to the W 4f_7p/2_ and W 4f_5p/2_ levels of the W–S bonds, while peaks located at 35.6 and 37.8 eV corresponded to the W 4f_7/2_ and W 4f_5/2_ levels of W–O bonding. The core-level XPS profiles of the WS_2_ NFs and WS_2_–WC–WO_3_ NH spheres are shown in Additional file 1: Figures S2 and S3, respectively. Both the samples showed high-resolution C 1 s and O 1 s peaks. However, in the XPS profiles of the WS_2_ NFs, the oxygen peaks were detected because of the surface oxidation of WS_2_, while the carbon peaks were attributed to the graphitic TAT used in the synthesis process. This is consistent with the XRD and Raman spectroscopy results. The S 2p, N 1 s, and O 1 s PS profiles of the WS_2_ NFs are shown in Additional file 1: Figure S2a–c. In contrast, the oxygen and carbon peaks in the XPS profile of the WS_2_–WC–WO_3_ NH spheres indicate the co-existence of WC and WO_3_. The co-existence of WC and WO_3_ in the WS_2_–WC–WO_3_ NH composite was also indicated by the XRD and Raman spectroscopy results. To understand the complex peaks of the other elements on the surface of the materials, the S 2p, N 1 s, O 1 s and C 1 s peaks of the WS_2_–WC–WO_3_ NH composite were deconvoluted, as shown in Additional file 1: Figure S3a–d. The S 2p peaks of the WS_2_ NFs and WS_2_–WC–WO_3_ NH spheres were also significantly different. The S 2p peak of the WS_2_–WC–WO_3_ NH composite could be deconvoluted into three main peaks located at approximately 161.9, 163.2, and 164.5 eV corresponding to the S 2p_3/2_ and S 2p_1/2_ orbitals of divalent sulfide ions and C–S=C bonding, respectively, as shown in Additional file 1: Figure S3a. In contrast, as shown in Additional file 1: Figure S2a the S 2p peak of the WS_2_ NFs could be deconvoluted into two main peaks centered at 161.6 and 163 eV corresponding to the S 2p_3/2_ and S 2p_1/2_ orbitals of divalent sulfide ions, respectively [50]. The N 1 s XPS peak of the WS_2_–WC–WO_3_ NH composite could be deconvoluted into two components centered at approximately 400.1 and 402.8 eV, corresponding to free nitrogen and hydrogen bonding amine, respectively (Additional file 1: Figure S3b) [51]. The O 1 s peak of the WS_2_–WC–WO_3_ NH composite could be deconvoluted into –O– and –OH peaks, which were attributed to surface contamination and O^2-^ combined with W ions, respectively [52]. The C1s peak could be deconvoluted into several peaks, as shown in Additional file 1: Figure S3d. We focused on the two strongest peaks centered at approximately 284.8 and 286 eV corresponding to the sp^2^ and sp^3^ hybridization of the C–C or C–H bonds, respectively [53].

**Fig. 5:**
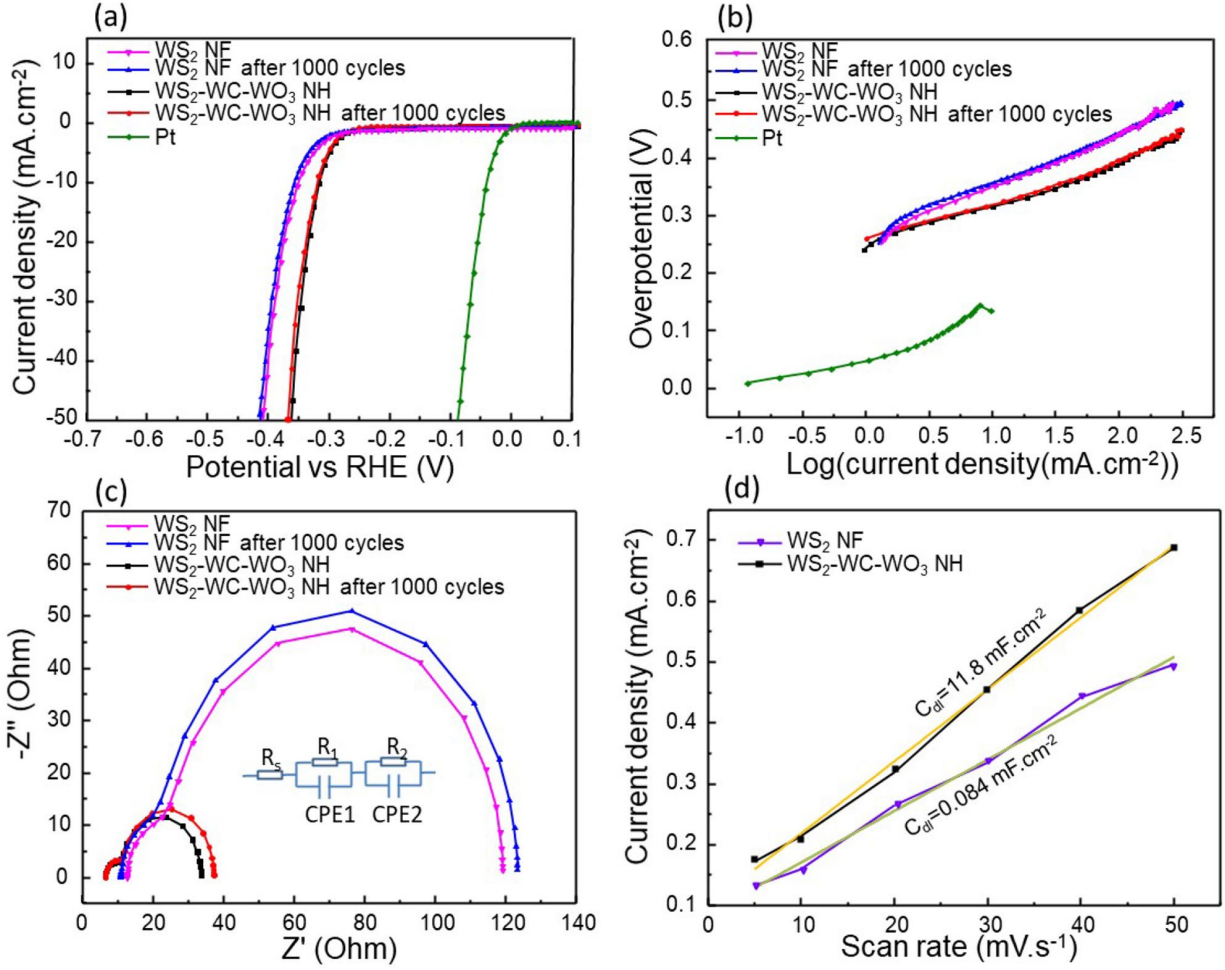
HER catalytic activities of the WS_2_ NF, WS_2_ NF(AT), WS_2_–WC–WO_3_ NH and WS_2_–WC–WO_3_ NH (AT) samples. **a** Polarization curves of the WS_2_ NF, WS_2_ NF(AT), WS_2_–WC–WO_3_ NH, and WS_2_–WC–WO_3_ NH (AT) samples and commercial Pt/C at a scan rate of 5 mV s^−1^, **b** Tafel plots of the WS_2_ NF, WS_2_ NF(AT), WS_2_–WC–WO_3_ NH, and WS_2_–WC–WO_3_ NH (AT) samples and standard Pt/C, **c** EIS results with the inset showing an equivalent circuit and **d** double-layer capacitances of the WS_2_ NF and WS_2_–WC–WO_3_ NH samples

The HER performances of the WS_2_ NFs and WS_2_–WC–WO_3_ NH spheres were carefully investigated using a three-electrode system (Fig. 5). The HER performance of the WS_2_ NFs was highly consistent with that obtained in our previous study [41]. The Tafel slope, impedance, and double-layer capacitance of the WS_2_ NFs were approximately 82 mV dec^−1^, 95 Ω, and 0.08 mF cm^−2^, respectively. To drive a cathodic current density of − 10 mA cm^−2^, an overvoltage of − 352 mV was employed. The stability of WS_2_ NFs was tested for 1000 cycles, the polarization curves of tested samples was carried out. The HER performance of WS_2_ NFs after test (AT) was presented in Fig. 5. After stability test, the Tafel slope of WS_2_ NFs AT was increased from 82 mV dec^−1^ to 83.5 mV dec^−1^. The data indicates that WS_2_ NFs is quite stable in acid media for HER application with a negligible shift after stability test. We also investigated the HER performance of the WS_2_–WC–WO_3_ NH spheres before and after test (WS_2_–WC–WO_3_ NH (AT))-the stability test using cyclic voltammetry (1000 cycles), which was denoted as WS_2_–WC–WO_3_ NH (AT). The electrocatalytic activity of the WS_2_–WC–WO_3_ NH composite toward the HER was measured using a three-electrode system in a standard acidic medium of 0.5 M H_2_SO_4_. The stability the WS_2_–WC–WO_3_ NH composite was evaluated over the overpotential range of − 0.3 to − 0.5 V for 1000 cycles, as shown in Additional file 1: Figure S5b. Figure 5a shows the polarization curves of the WS_2_ NF, WS_2_–WC–WO_3_ NH, and WS_2_–WC–WO_3_ NH (AT) samples and a standard platinum electrode. After 1000 cycles, the overpotential of the WS_2_–WC–WO_3_ NH sample decreased slightly as compared to the initial value, indicating the outstanding durability of the sample. At the current density of − 10 mA cm^−2^, the overpotential of the WS_2_–WC–WO_3_ NH sample was − 0.312 mV, which is much better than that of the WS_2_ NFs (− 352 mV). Therefore, the WS_2_–WC–WO_3_ NH composite is a promising material for water dissociation as compared to the WS_2_ NFs. To further evaluate the HER performances of the WS_2_–WC–WO_3_ NH and WS_2_–WC–WO_3_ NH (AT) samples in acidic media, their Tafel slopes were plotted, as shown in Fig. 5b. The Tafel slopes of the WS_2_–WC–WO_3_ NH and WS_2_–WC–WO_3_ NH (AT) samples were 59 and 60 mV dec^−1^, respectively. This confirms that the stability of the WS_2_–WC–WO_3_ NH material was much better than that of the WS_2_ NFs (82 mV dec^−1^). Figure 5c shows the electronic properties of the materials before and after the cyclic voltammetry (CV) test. The equivalent circuit shown in the inset of Fig. 5c was composed of constant phase elements and charge-transfer resistances. The charge-transfer resistances included the wire connection (R_s_), the resistance between the electrocatalysts and solution (R_1_), and the resistance between the electrocatalysts and GCE (R_2_). The fitting values are listed in Table 1. The connection showed a resistance of 6.44–6.47 Ω. More importantly, the charge transfer resistance of the WS_2_–WC–WO_3_ NH composite was approximately 22.94 Ω, indicating that the electron conduction on the surface of the hollow composite was better than that of the pure WS_2_ NFs (94.36 Ω). This means that the conductivity of the WS_2_–WC–WO_3_ NH composite was much higher than that of the WS_2_ NFs. The double-layer capacitance (C_dl_) of the WS_2_–WC–WO_3_ NH composite and WS_2_ NFs was calculated via CV scanning at different scan rates of 5, 10, 20, 30, 40, and 50 mV s^−1^. The CV curves were obtained within the potential window of 0–0.2 V, where no Faradaic current was observed. The CV scans of the WS_2_ NF and WS_2_–WC–WO_3_NH samples at various scan rates are shown in Additional file 1: Figures S4a and S5a, respectively. Figure 5d shows that the C_dl_ of the WS_2_–WC–WO_3_ NH and WS_2_ NF samples were approximately 11.8 mF cm^−2^ and 0.084 mF cm^−2^, respectively. The higher C_dl_ of the WS_2_–WC–WO_3_ NH composite can be attributed to its hollow sphere structure, owing to which it had a larger active surface and was robust to surface depletion during the electrochemical process. Additional file 1: Table S1 presents this work results and previous data. It indicates that WS_2_–WC–WO_3_ NH is a prominent catalyst material which could be applied for HER application.

**Table 1:** The fitting values of charge-transfer resistances (Rs), the resistance between the electrocatalysts and solution (R1), and the resistance between the electrocatalysts and GCE (R2) of samples

	R_s_ (Ω)	R_1_ (Ω)	R_2_ (Ω)
WS_2_–WC–WO_3_ NH	6.44	22.94	4.271
WS_2_–WC–WO_3_ NH (AT)	6.47	25.91	4.89
WS_2_ NF	12.69	94.36	12.22
WS_2_ NF (AT)	12.74	98.25	13.01

Another important parameter affecting the electrochemical performance of a material is its electrochemical active surface area (ECSA). ECSA represents the area of an electrode material that is accessible to the solution for electron transfer or charge storage. The ECSA of an electrode can be calculated using the following equations [54, 55]:

1 $$\text{ECSA}=\frac{{C}_{dl}}{{C}_{s}}$$

where C_s_ is the capacitance per unit area of the smooth planar surface of the material. Here we used a general specific capacitance of C_s_ = 0.04 mF/cm^2^ in 0.5 M H_2_SO_4_. The C_dl_ values of the WS_2_–WC–WO_3_NH and WS_2_ NF samples were calculated to be 11.8 and 0.084 mF cm^−2^, respectively. By substituting the values of C_dl_ and C_s_ in Eq. (1), the ECSA values of the WS_2_–WC–WO_3_ NH and WS_2_ NF samples were calculated to be 295 and 2.1, respectively. These values indicate that the WS_2_–WC–WO_3_ NH composite showed better charge transfer or charge storage than the WS_2_ NFs.

The turnover frequency (TOF) of H_2_ is the number of H_2_ molecules generated per active site per unit time. The TOF of H_2_ produced by the WS_2_ NFs and WS_2_–WC–WO_3_ NH spheres was calculated using the following formula [56, 57].

2 $$\text{TOF}=\frac{jA}{4nF}$$

where j is the current density (A cm^− 2^), A is the surface area of the working electrode (cm^2^), n is the number of moles of the catalyst loaded onto the working electrode, and F is the faraday constant (F = 96485.3329 C mol^− 1^). The TOFs of H_2_ production at η = −312 mV and − 352 mV (current density measured = 10 mA cm^− 2^) for the WS_2_ NF and WS_2_–WC–WO_3_ NH samples were 0.08 and 0.02 s^− 1^, respectively, and the corresponding results are shown in Additional file 1: Figures S4b and S5c.

**Fig. 6:**
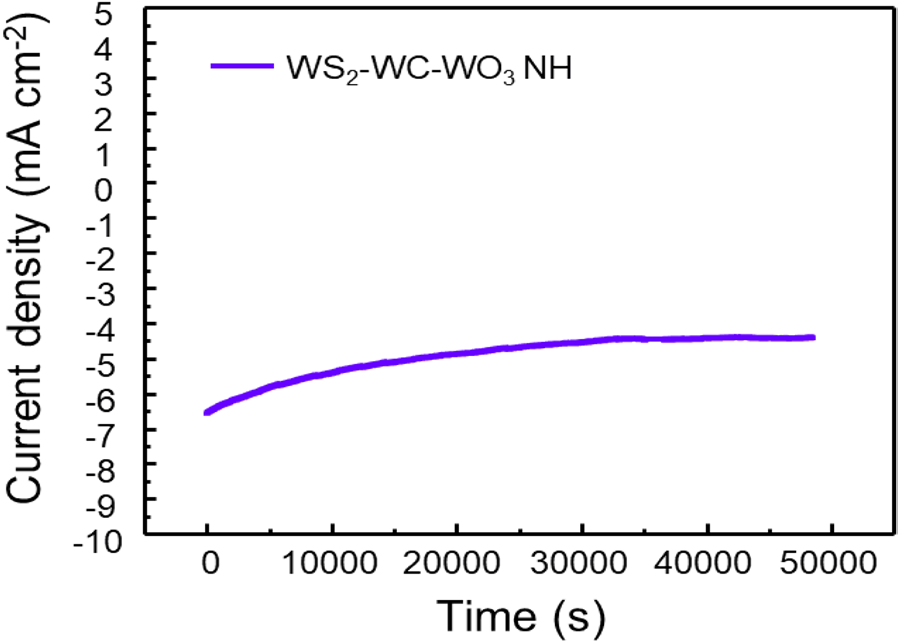
Time-dependent current density of the WS_2_–WC–WO_3_NH spheres at − 0.33 V for 50,000 s

Finally, the stability of the WS_2_–WC–WO_3_ NH spheres was also investigated by prolonged electrolysis at a constant overpotential of − 0.33 V. As can be observed from Fig. 6, the sample showed excellent stability in a 0.5 M H_2_SO_4_ solution for 50,000 s. The results indicate that the composite prepared in this study is a promising material for electrochemical applications.

## Conclusions

In this study, we developed r a facile and low-cost procedure to prepare a novel composite of WS_2_ (TMD), WC (TMC), and WO_3_ (TMO) (WS_2_–WC–WO_3_ NH) using the conventional solvothermal technique. Interestingly, the hybrid material was doped in-situ with nitrogen. The electrochemical measurements of the WS_2_–WC–WO_3_NH composite were also carried out in an acidic medium to evaluate its electrocatalytic performance for the HER. The results indicated that the WS_2_–WC–WO_3_ NH composite exhibited extraordinary electrocatalytic properties as compared to the pure WS_2_ NFs. This improvement can be attributed to the presence of WC and WO_3_, which exhibited a synergistic effect with WS_2_ and endowed the composite with various advantages of TMC and TMO materials. Moreover, the nitrogen doping significantly increased the electrical conductivity of the hybrid material. The Tafel slopes of the WS_2_–WC–WO_3_ NH composite before and after the CV test (1000 cycles) were 59 and 60 mV dec^−1^, respectively. These results demonstrate the excellent performance and stability of the WS_2_–WC–WO_3_ NH catalyst for the HER. Therefore, the WS_2_–WC–WO_3_ NH composite prepared in this study is a promising alternative to expensive noble metal catalysts for the HER and other electrochemical applications.

## Supplementary Information

**Additional file 1: Figure S1.** EDS map images of WS2-WC-WO3 NH spheres showing the spatial elemental distribution for W, S, O, C and N atoms. **Figure S2.** High-resolution (**a**) S 2p, (**b**) N 1s, and (**c**) O1s XPS profiles of the WS2 NFs. **Figure S3.** High-resolution (**a**) S 2p, (**b**) N 1s, (**c**) O1s, and (**d**) C1s XPS profiles of the WS2-WC-WO3 NH spheres. **Figure S4.** (**a**) CV curves and (**b**) TOF of the WS2 NFs. **Figure S5.** CV curves of the WS2-WC-WO3 NH composite at various scan rates (**a**) before and (**b**) after 1000 cycles and (**c**) TOF of the WS2-WC-WO3 NH composite. **Table S1.** Comparison of other electrocatalysts previously reported in HER.

## Data Availability

The datasets used and/or analyzed during the current study are available from the corresponding author upon reasonable request.

## Abbreviations

NH: Nano-hollow
NF: Nanoflower
HER: Hydrogen evolution reaction
TMD: Transition metal dichalcogenide
TMO: Transition metal oxide
TMC: Transition metal carbide
FE-SEM: Field emission scanning electron microscopy
XRD: X-ray diffraction
XPS: X-ray photoelectron spectroscopy
CV: Cyclic voltammetry
EIS: Electrochemical impedance spectroscopy
AMT: Ammonium meta-tungstate
TAT: Thioacetamide
DI: De-ionized
GCE: Glassy carbon electrode
LSV: Linear sweep voltammetry
RHE: Reference hydrogen electrode
ECSA: Electrochemical active surface area
TOF: Turnover frequency
