# Correction to: WS_2_–WC–WO_3_ nano‑hollow spheres as an efficient and durable catalyst for hydrogen evolution reaction

**DOI:** 10.1186/s40580-021-00284-5

**Published:** 2021-10-19

**Authors:** Tuan Van Nguyen, Ha Huu Do, Mahider Tekalgne, Quyet Van Le, Thang Phan Nguyen, Sung Hyun Hong, Jin Hyuk Cho, Dung Van Dao, Sang Hyun Ahn, Soo Young Kim

**Affiliations:** 1 School of Chemical Engineering and Materials Science, Chung-Ang University, 84 Heukseok-ro, Dongjak-gu, Seoul, 06974 Republic of Korea; 2 Department of Materials Science and Engineering, Institute of Green Manufacturing Technology, Korea University, 145 Anam-ro, Seongbuk-gu, Seoul, 02841 Republic of Korea; 3 Department of Chemical and Biological Engineering, Gachon University, Seongnam-si, Gyeonggi-do 13120 Republic of Korea

## Correction to: Nano Convergence (2021) 8:28 https://doi.org/10.1186/s40580-021-00278-3

Following publication of the original article [1], the affiliation of the authors was incorrectly published in the article. The affiliation which was shown in supplementary information is correct. This has been corrected with this erratum.
