# Supplementary material for: WS2–WC–WO3 nano-hollow spheres as an efficient and durable catalyst for hydrogen evolution reaction
Source: Nano Converg. 2021 Sep 20;8:28. doi: 10.1186/s40580-021-00278-3 (PMC8452812; doi:10.1186/s40580-021-00278-3)
Supplement: Supplementary file 1 — Additional file 1: Figure S1. EDS map images of WS2-WC-WO3 NH spheres showing the spatial elemental distribution for W, S, O, C and N atoms. Figure S2. High-resolution (a) S 2p, (b) N 1s, and (c) O1s XPS profiles of the WS2 NFs. Figure S3. High-resolution (a) S 2p, (b) N 1s, (c) O1s, and (d) C1s XPS profiles of the WS2-WC-WO3 NH spheres. Figure S4. (a) CV curves and (b) TOF of the WS2 NFs. Figure S5. CV curves of the WS2-WC-WO3 NH composite at various scan rates (a) before and (b) after 1000 cycles and (c) TOF of the WS2-WC-WO3 NH composite. Table S1. Comparison of other electrocatalysts previously reported in HER. [file 40580_2021_278_MOESM1_ESM.docx]

**Supporting Information**

**WS_2_-WC-WO_3_ nano hollow spheres as an efficient and durable catalyst for hydrogen evolution reaction**

*Tuan Van Nguyen*^1^*, Ha Huu Do*^1^*, Mahider Tekalgne*^1^*, Quyet Van Le*^2^*, Thang Phan Nguyen*^3^*, Sung Hyun Hong*^2^*, Jin Hyuk Cho*^2^*, Dung Van Dao*^2^*, Sang Hyun Ahn*^1^*^*^*^s^*, Soo Young Kim*^2^*^*^*

^1^School of Chemical Engineering and Materials Science, Chung-Ang University, 84 Heukseok-ro, Dongjak-gu, Seoul, 06974, Republic of Korea

^2^Department of Materials Science and Engineering, Institute of Green Manufacturing Technology, Korea University, 145 Anam-ro, Seongbuk-gu, Seoul, 02841, Republic of Korea

^3^Department of Chemical and Biological Engineering, Gachon University, Seongnam-si, Gyeonggi-do, 13120, Republic of Korea


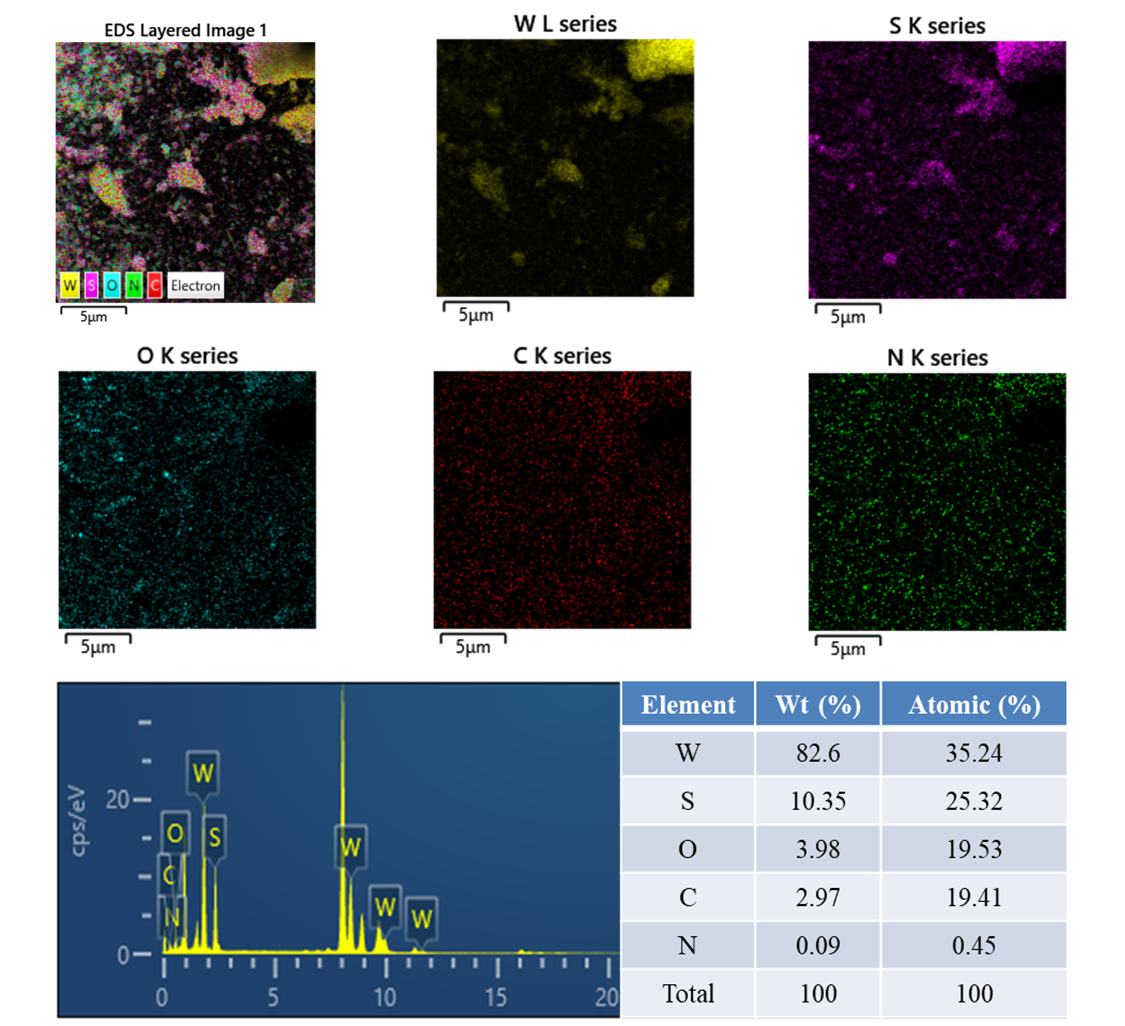


**Figure S1**. EDS map images of WS_2_-WC-WO_3_ NH spheres showing the spatial elemental distribution for W, S, O, C and N atoms.


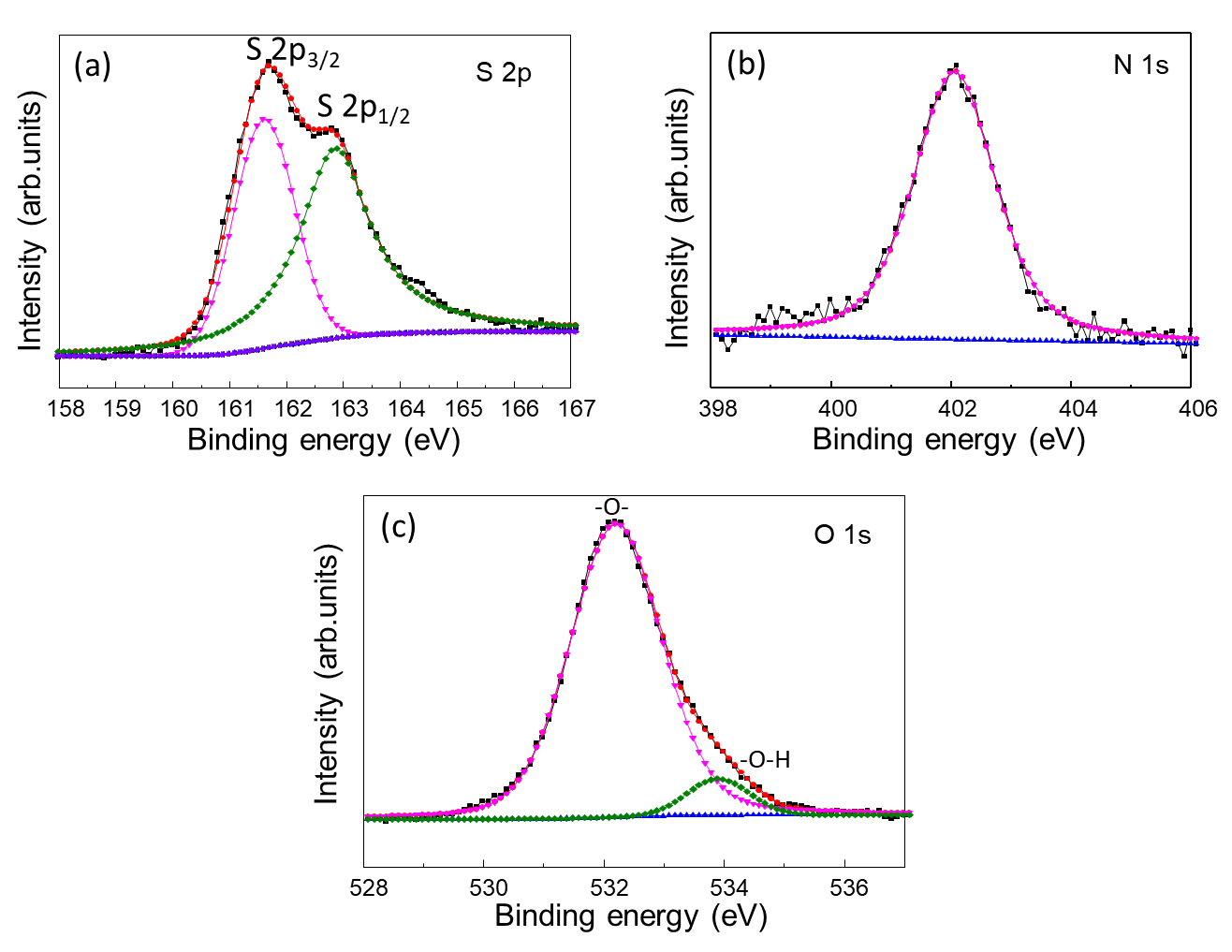


**Figure S2**. High-resolution (a) S 2p, (b) N 1s, and (c) O1s XPS profiles of the WS_2_ NFs.


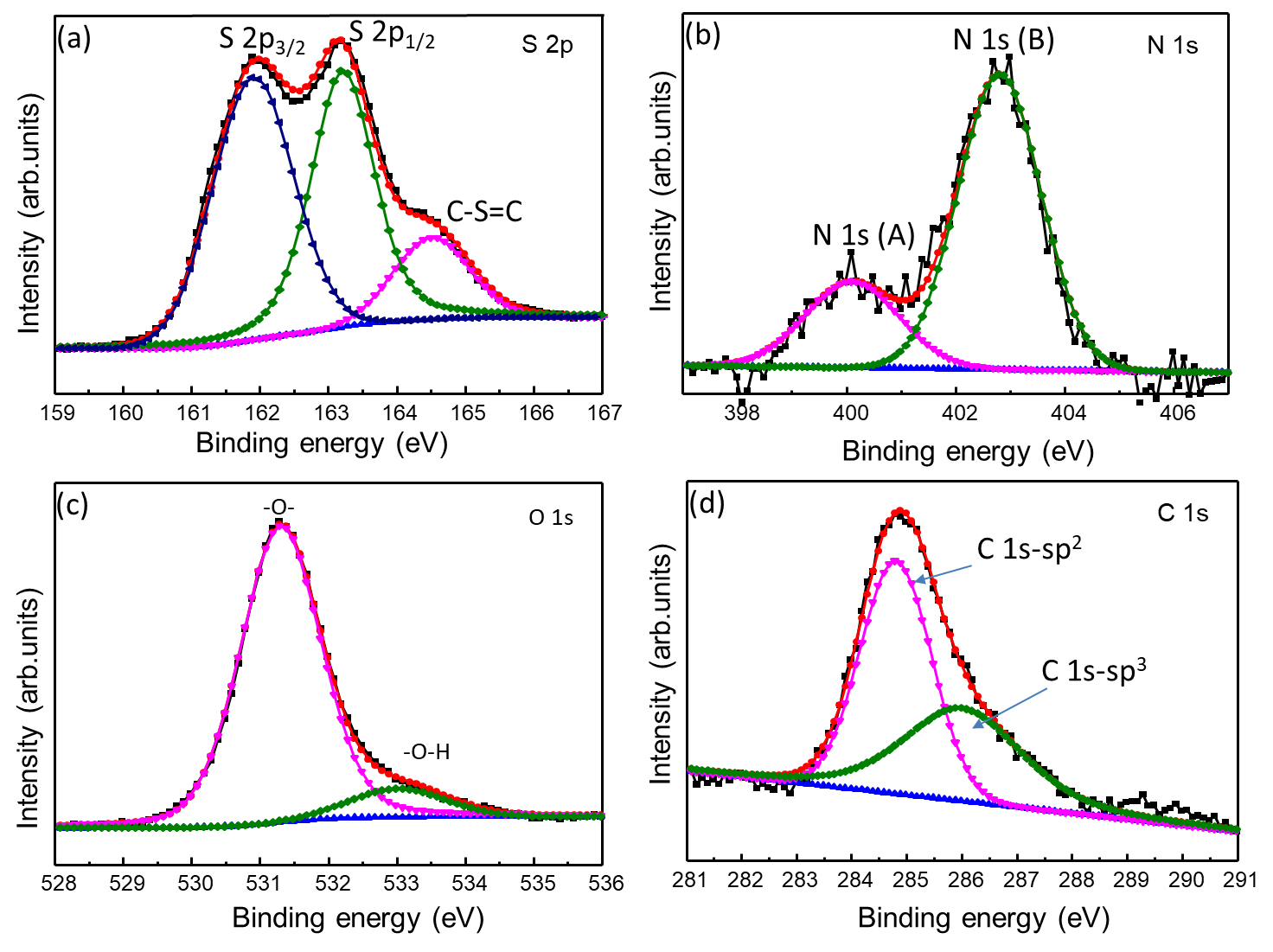


**Figure S3**. High-resolution (a) S 2p, (b) N 1s, (c) O1s, and (d) C1s XPS profiles of the WS_2_-WC-WO_3_ NH spheres.


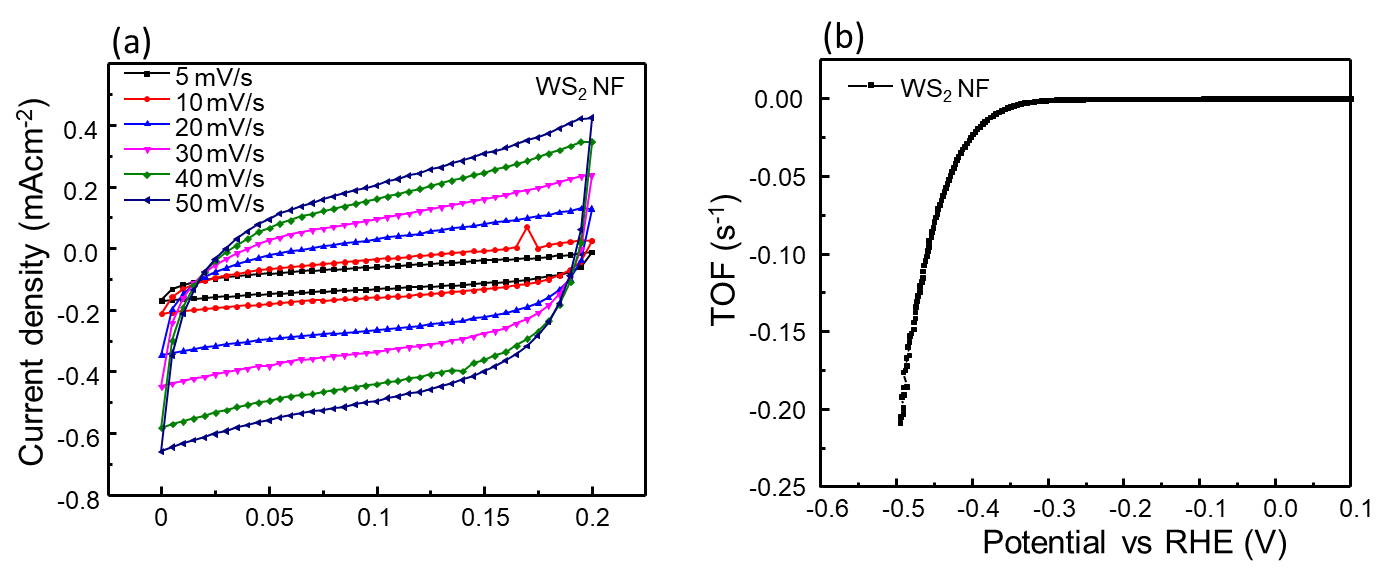


**Figure S4**. (a) CV curves and (b) TOF of the WS_2_ NFs.


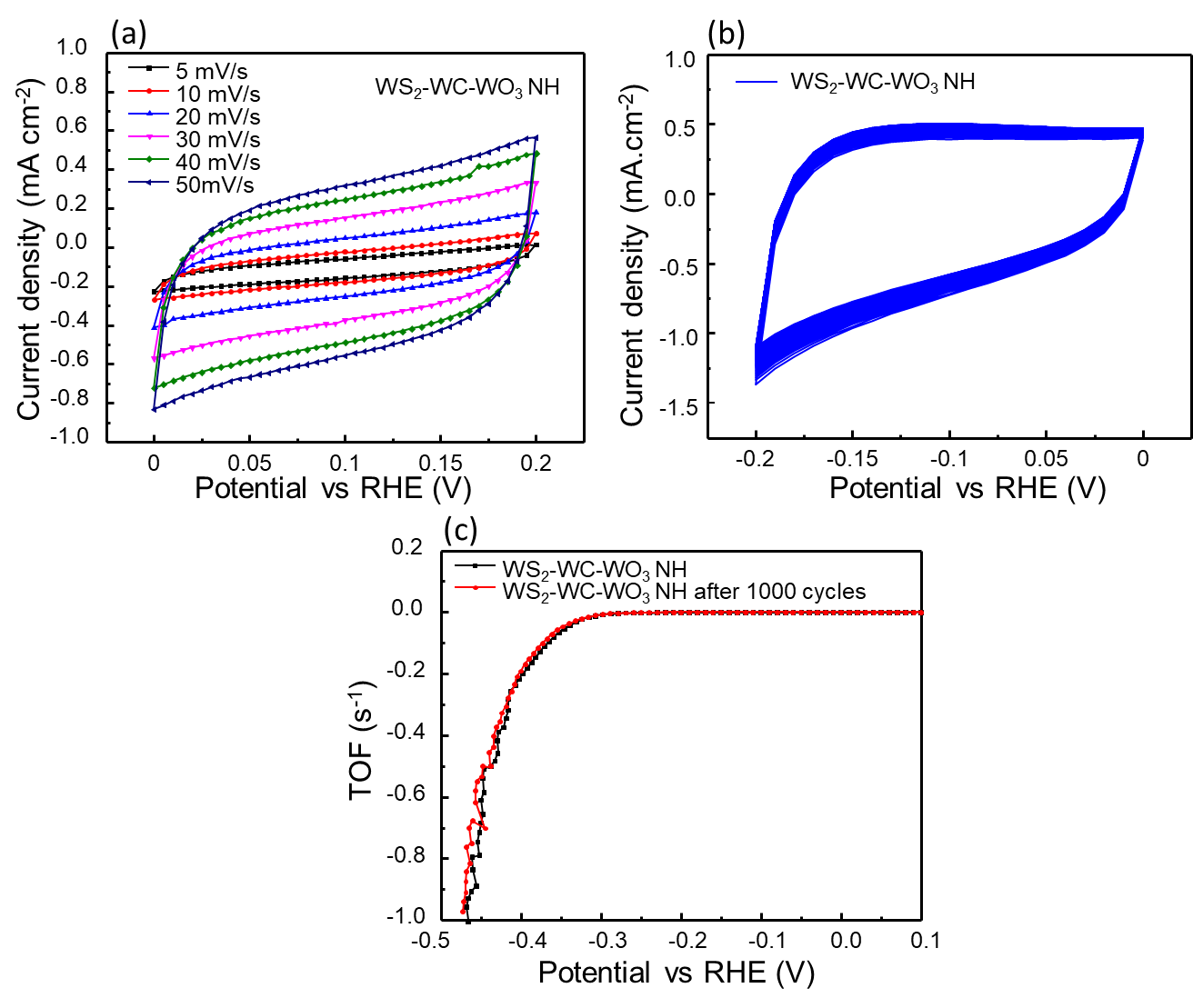


**Figure S5**: CV curves of the WS_2_-WC-WO_3_ NH composite at various scan rates (a) before and (b) after 1000 cycles and (c) TOF of the WS_2_-WC-WO_3_ NH composite.

The value of ECSA and C_dl_ were interpreted in our previous study[1]. ECSA calculation was performed following equation:

$ECSA=\frac{C_{dl}}{C_{s}}$ (1)

where C_dl_ is double layer capacitance measured through cyclic test which is equal to the slope of the fitted line Figure 5 (d); C_s_ is the capacitance of the sample of an atomically smooth planar surface of material per unit area under identical electrolyte conditions. Here, we use general specific capacitance of C_s_ = 0.04 mF/cm^2^ in 0.5M H_2_SO_4_ based on previous reported value [2]. The cyclic test to calculate C_dl_ was measured from 0.2 V to 0 V at different scan rate from 5, 10, 20, 30, 40 to 50 mV s ^−1^. The C_dl_ of WS_2_ nanoflowers and WS_2_-WC-WO_3_ NH were calculated of 0.084 mF cm^−2^ and 11.8 mF cm^−2^, respectively. By applying the value of C_dl_ to equation (1), we obtained ECSA of 2.1 and 295 for WS_2_ nanoflower and WS_2_-WC-WO_3_ NH, accordingly.

**Table S1**. Comparison of other electrocatalysts previously reported in HER

| Catalysts | Onset potential (mV) | Tafel sloped  (mV dec^-1^) | Double layer capacitance C_dl_ (mFcm^-2^) | Reference |
| --- | --- | --- | --- | --- |
| N doped WS2 | 197 (100mA/cm^2^) | 69.69 | 23.5 | [3] |
| WS2 hollow | 125.2 | 60 | 1.3 | [1] |
| W2C@WS2 | 180 | 55.4 | 12.28 | [4] |
| WS2-rGO | 170 (10mA/cm^2^) | 52 |  | [5] |
| MoS2/WS2/rGO | 157 (10mA/cm^2^) | 44 |  | [6] |
| MoS2-WS2 | 129 (10mA/cm^2^) | 72 |  | [7] |
| WS2-TiO2 | 142 (10mA/cm^2^) | 120 | 35.2 | [8] |
| WS2/WO3 | 395 (10mA/cm^2^) | 50 |  | [9] |
| WS2-WC-WO3 | 312 | 59 | 11.8 | This work |

**Reference**

1 T. P. Nguyen, D. L. T. Nguyen, V.H. Nguyen, T.H. Le, Q. V. Ly, D.V. N. Vo, Q. V. Nguyen, H. S. Le, H. W. Jang & S. Y. Kim, Facile synthesis of WS_2_ hollow spheres and their hydrogen evolution reaction performance. Appl. Surf. Sci. **505**, 144574 (2020).

2 J. Hu, B. Huang, C. Zhang, Z. Wang, Y. An, D. Zhou, H. Lin, M. K. H. Leung & S. Yang, Engineering stepped edge surface structures of MoS_2_ sheet stacks to accelerate the hydrogen evolution reaction. Energy Environ. Sci. **10**, 593-603 (2017).

3 C. Sun, J. Zhang, J. Ma, P. Liu, D. Gao, K. Tao & D. Xue, N-doped WS_2_ nanosheets: a high-performance electrocatalyst for the hydrogen evolution reaction. Journal of Materials Chemistry A. **4**, 11234-11238 (2016).

4 T. P. Nguyen, S. Y. Kim, T. H. Lee, H. W. Jang, Q. Van Le & I. T. Kim, Facile synthesis of W_2_C@WS_2_ alloy nanoflowers and their hydrogen generation performance. Appl. Surf. Sci. **504**, 144389 (2020).

5 J. Zhang, Q. Wang, L. Wang, X. a. Li & W. Huang, Layer-controllable WS_2_-reduced graphene oxide hybrid nanosheets with high electrocatalytic activity for hydrogen evolution. Nanoscale. **7**, 10391-10397 (2015).

6 H. J. Lee, S. W. Lee, H. Hwang, S. I. Yoon, Z. Lee & H. S. Shin, Vertically oriented MoS_2_/WS_2_ heterostructures on reduced graphene oxide sheets as electrocatalysts for hydrogen evolution reaction. Materials Chemistry Frontiers. **5**, 3396-3403 (2021).

7 D. Vikraman, S. Hussain, K. Akbar, L. Truong, A. Kathalingam, S.-H. Chun, J. Jung, H. J. Park & H.-S. Kim, Improved hydrogen evolution reaction performance using MoS_2_–WS_2_ heterostructures by physicochemical process. ACS Sustainable Chemistry & Engineering. **6**, 8400-8409 (2018).

8 S. Liu, Y. Xu, D. Chanda, L. Tan, R. Xing, X. Li, L. Mao, N. Kazuya & A. Fujishima, Ultrathin WS_2_ nanosheets vertically aligned on TiO2 nanobelts as efficient alkaline hydrogen evolution electrocatalyst. Int. J. Hydrogen Energy. **45**, 1697-1705 (2020).

9 X. Shang, Y. Rao, S.-S. Lu, B. Dong, L.-M. Zhang, X.-H. Liu, X. Li, Y.-R. Liu, Y.-M. Chai & C.-G. Liu, Novel WS_2_/WO_3_ heterostructured nanosheets as efficient electrocatalyst for hydrogen evolution reaction. Mater. Chem. Phys. **197**, 123-128 (2017).
